# Interoceptive hypersensitivity and interoceptive exposure in patients with panic disorder: specificity and effectiveness

**DOI:** 10.1186/1471-244X-6-32

**Published:** 2006-08-16

**Authors:** Kiyoe Lee, Yumiko Noda, Yumi Nakano, Sei Ogawa, Yoshihiro Kinoshita, Tadashi Funayama, Toshiaki A Furukawa

**Affiliations:** 1 Department of Psychiatry and Cognitive-Behavioral Medicine, Nagoya City University Graduate School of Medical Sciences, Mizuho-cho, Mizuho-ku, Nagoya 467–8601, Japan

## Abstract

**Background:**

Interoceptive exposure has been validated as an effective component of cognitive behavioral therapy (CBT) for the treatment of panic disorder but has hitherto received little research attention. We examined the effectiveness of various interoceptive exposure exercises using the Body Sensations Questionnaire (BSQ) (Chambless et al., 1984).

**Methods:**

We first performed an exploratory principal factor analysis of all the items contained in the BSQ to obtain meaningful dimensions of interoceptive fears. Next, we examined the correlations between each interoceptive exposure task's degree of similarity to panic attacks and each BSQ factor and then examined whether the BSQ factor scores decreased in comparison with the baseline values when the corresponding exposure tasks were successfully completed by the subjects.

**Results:**

The factor analyses revealed four factors, which we named "pseudoneurological fears", "gastrointestinal fears", "cardiorespiratory fears" and "fears of dissociative feelings." Among the nine interoceptive exposure tasks, 'hyperventilation', 'shaking head', 'holding breath' and 'chest breathing' were considered to reproduce pseudoneurological symptoms, 'breathing through a straw' was considered to reproduce gastrointestinal symptoms, and 'spinning' was considered to reproduce both pseudoneurological and dissociative symptoms; none of the interoceptive exercises were found to reproduce cardiorespiratory symptoms. Among each group of patients for whom 'hyperventilation', 'holding breath', 'spinning' or 'chest breathing' was effective, a significant improvement in the BSQ pseudoneurological fears factor scores was observed. On the other hand, no significant difference between the baseline and endpoint values of the BSQ gastrointestinal fears or the BSQ fears of dissociative feelings factor scores were observed among the patients for whom 'spinning' or 'breathing through a straw' was effective.

**Conclusion:**

Several interoceptive exposure tasks were particularly effective in reducing pseudoneurological fears. New interoceptive tasks, especially tasks related to cardiorespiratory and dissociative feelings, are needed.

## Background

Panic disorder is a common but disabling anxiety disorder. The chief defining criteria for panic disorder are recurrent unexpected panic attacks, persistent concern about having an additional attack, and worry about the implications of an attack or its consequences [1]. The essential feature of a panic attack is an intense fear or discomfort in the absence of real danger, accompanied by somatic or cognitive symptoms (e.g. palpitations, sweating, trembling, and fear of losing control).

One theoretical perspective of panic disorders maintains that the central component of this disorder is hypersensitivity to physical sensations. Panic disorder can then be conceptualized as a phobic fear of physical sensations caused by traumatic conditioning from unexpected panic attacks and catastrophic misinterpretations of physical sensations [2]. Goldstein and Chambless [3] labeled this fear of experiencing anxiety or panic attacks as "fear of fear". Several other conceptualizations of panic disorder emphasize the role that the fear of physical sensations serves as an important maintaining factor for this disorder [2-4]. Each of these theories ascribe importance to "fear of fear" as a maintaining factor.

In keeping with this perspective, cognitive-behavioral therapy (CBT) for the treatment of panic disorder consists of three primary components: first, strategies to reduce physiological arousal (e.g., relaxation training or breathing retraining); second, cognitive therapeutic procedures to change catastrophic misinterpretations of panic-related sensations and assist in the gathering of potentially corrective information; and third, exposure procedures to let the patient experience the feared stimuli. Exposure procedures have two forms: exposure to environmental situations that each patient fears, termed in vivo exposure; and exposure to exercises that evoke the physical sensations associated with panic attacks (e.g., hyperventilation, shaking head and body tension), termed interoceptive exposure. In particular, interoceptive exposure is thought to be unique to the treatment of panic disorder and to focus directly on the patient's fear of physical sensations. Individuals with panic disorder responded more strongly to symptom induction exercises than did controls [5]. Deliberate repeated exposure to physical sensations is thought to facilitate the habituation of anxiety in patients with panic disorder [6]. Several studies have proven the efficacy of interoceptive exposure [7-10], but this procedure has not yet been studied as much as in vivo exposure has been, and no study has examined the effectiveness of each interoceptive exposure exercise.

The present study examined the efficacy of interoceptive exposure using the Body Sensations Questionnaire (BSQ) [11], which covers the fear of panic-related physical sensations. In particular, we examined (1) whether each interoceptive exposure exercise reproduces specific feared physical sensations and (2) if so, whether the successful completion of the interoceptive exposure task reduces the corresponding fear of physical sensations.

## Methods

### Subjects

A consecutive series of 96 patients were recruited into the group CBT program for panic disorder at the Department of Psychiatry, Nagoya City University Hospital, between October 2001 and March 2005; all of the patients met the following entry criteria: (a) principal Axis I diagnosis of panic disorder with or without agoraphobia according to the DSM-IV(Diagnostic and Statistical Manual of Mental Disorders, Fourth Edition) criteria, as assessed by the Structured Clinical Interview for DSM-IV [12]; (b) absence of current psychosis, bipolar disorder and substance-use disorder; (c) highly motivated to undergo CBT; and (d) free from benzodiazepine-use prior to CBT entry, since these drugs interact negatively with exposure treatments [13-15]. Use of antidepressants was permitted throughout the CBT period because these drugs do not interfere with CBT treatments [16]. The patients provided their written informed consent after receiving a full explanation of the study's purpose and procedures. The study protocol was approved by the Ethics Committee of Nagoya City University Graduate School of Medical Sciences.

### Procedure

#### 1. CBT

We followed the established CBT treatment manual for panic disorder, written by Andrews et al. [17]. Treatments were conducted in groups of three to four patients led by one principal therapist and one co-therapist and were scheduled to last 120 minutes. Each session was held once a week, for a total of 10 sessions.

The first two sessions included psychoeducation about the nature of anxiety, panic and agoraphobia and provided a rationale for and training in breathing retraining. We placed a stronger emphasis on slow-breathing techniques than on relaxation training. From the third session onwards, cognitive restructuring – including both in vivo exposure and interoceptive exposure – were introduced, and the patients were asked to try to formulate rational thoughts and to perform self-exposure tasks to reproduce both external and interoceptive phobic cues during and between sessions.

#### 2. Interoceptive exposure

Interoceptive exposure is specifically aimed at reducing the fear of physical sensations. Patients are requested to engage in a series of exercises that are supposed to produce sensations similar to those that occur during a panic attack. After explaining the rationale for interoceptive exposure, nine interoceptive exercises were covered during a therapist-led session; the nine exercises were designed to elicit a range of physical sensations (1. hyperventilation, 2. shaking head, 3. putting the head between the legs, 4. step-ups, 5. holding breath, 6. body tension, 7. spinning, 8. breathing through a straw, and 9. chest breathing). After completing all of these exercises, the patients were asked to score (on a scale from 0 to 100) three different aspects of the sensations aroused by each interoceptive task: (1) the level of discomfort produced by the sensations, (2) the degree to which the sensations were similar to those experienced during a panic attack and (3) the level of fear produced.

Next, a hierarchy was constructed for the interoceptive exercises. First, the patients selected exercises that produced symptoms with ratings of at least 30 on the 0–100 point scale of similarity to their own panic attacks. Each patient therefore selected a different set of interoceptive tasks. Then, the patients ranked the selected exercises in the order of the level of fear they produced. That is, out of the selected exercises, the patients ranked the exercise producing the lowest level of fear as number 1. The exercise with the next highest level of fear was ranked as numbered 2, and so on. Patients were instructed to perform at least one exercise each day, beginning with number 1 on their list, until they had completed all the exercises and experienced a reduction in their level of fear. They were also instructed to record the level of fear evoked during each practice. Within-session practices were consolidated by the between-session assignments.

### Measures and rating times

The BSQ [11] was used to obtain a self-report of the fear evoked by panic-related physical sensations. The scale contains 17 items concerning the degree to which patients fear somatic symptoms commonly associated with panic attacks (e.g., heart palpitations, dizziness, etc). Items are rated on a five-point scale ranging from '1 = not frightened or worried by this sensation' to '5 = extremely frightened by this sensation.' The total score therefore ranges between 17 and 85. The reliability and validity of the Japanese version of the BSQ have been established [18].

Patients completed the BSQ as part of a standard battery of questionnaires, including the Fear Questionnaire (FQ; [19]) and the Agoraphobic Cognitions Questionnaire (ACQ; [11]), given at week 0 (session 1; baseline), 12 (session10; endpoint), 24 (3-month follow-up) and 60 (1-year follow-up). In addition, a clinician used the Panic Disorder Severity Scale (PDSS; [20]) to evaluate each patient at the study's baseline and endpoint.

### Analyses

The following analyses were performed to examine whether interoceptive exposure had any effect on patient anxiety regarding feared physical sensations. All statistical analyses were performed using SPSS, version 11.5 [21].

#### 1. Factor analyses of BSQ

Factor analysis can reduce the number of meaningful dimensions of interoceptive fears, thereby increasing the internal consistency reliability for the obtained factor scores and avoiding multiple comparisons and type I errors in subsequent analyses.

We first submitted all 17 items of the BSQ to exploratory principal factor analysis, using Promax rotation to extract factors that were relevant to the physical sensations associated with panic attacks. As the criterion to determine the number of factors, an Eigenvalue of 1.0 or greater was used (Kaiser Criterion). The appropriate number of required factors was also assessed using a scree plot. An item was considered to load onto a factor if its factor loading score exceeded 0.35. A factor score for each patient was automatically generated using the SPSS software.

The internal consistency reliability of the obtained factors was examined by calculating the Cronbach alpha coefficients. Values greater than 0.75 are generally considered to indicate adequate reliability.

#### 2. Correlations between degree of similarity of interoceptive exposure tasks and BSQ factors

Next, to determine which interoceptive exposure exercises reflected which interoceptive fears, we examined the Spearman rank correlations between the degree of similarity to panic attacks for each interoceptive exposure task and each BSQ factor at baseline. The degree of similarity reflected the patient's subjective judgment as to how similar the sensations aroused by each exercise were to their own panic sensations at the time when they first performed each exposure task.

#### 3. Change after successful interoceptive exposure

After each interoceptive exercise performed at home, the level of fear produced by the physical sensation was recorded; these records were reported at the next group session. When the level of fear evoked by a particular exercise was reduced by 30 points, compared with the first interoceptive exposure, that exercise was deemed to be successful, and we examined if the BSQ factor scores had decreased in comparison with the baseline. Because the BSQ produced an ordinal variable and was expected to show a non-normal distribution, the Wilcoxon test was used.

## Results

### Participants

**Figure 1:**
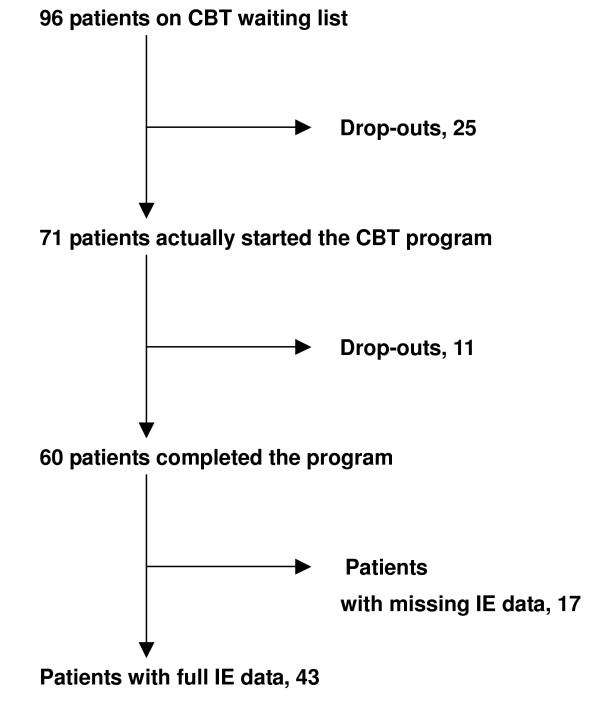
Subject enrolment.

Of the 96 patients, 25 decided not to start the CBT program because of symptom amelioration, geographical or time inconveniences, or pregnancy during the waiting period. Of the 71 patients who were actually enrolled in the program, 60 completed the treatment and 11 withdrew from the program because of an improvement experienced before the last session, a lack of improvement, the inconvenience of attending the sessions, or worsening depression. All patients who completed the CBT program assessed the similarity of each interoceptive exposure task to their own panic attacks, but, because of missing data in the medical records, only 43 complete records of the interoceptive exposure tasks were available for analysis. Figure 1 shows a flow diagram of the subjects and Table 1 shows their baseline demographic and clinical characteristics. No statistically significant differences were seen among the subgroups.

**Table 1:** Baseline demographic and clinical characteristics of the subjects

	CBT completers with full IE data (N = 43)	CBT completers with missing IE data (N = 17)	Drop-outs from treatment (N = 11)	Drop-outs during the waiting period (N = 25)	Between-group differences
Mean age (SD)	35.7 (11.3)	36.3 (12.1)	28.9 (4.65)	33.4 (12.4)	F(3,92) = 1.13 p = 0.34
Sex (Male, %)	26 (60%)	12 (70%)	8 (73%)	11 (49%)	χ^2 ^= 4.17 df = 3 p = 0.24
Panic disorder with agoraphobia (%)	37(86%)	15(88%)	9(82%)	19(76%)	χ^2 ^= 1.51 df = 3 p = 0.68
Mean age at onset (SD)	30.2(11.6)	25.7(8.53)	25.4(5.00)	28.8(13.0)	F(3,92) = 0.97 p = 0.41
PDSS	12.3 (4.75)	13.4 (4.91)	11.9 (5.30)	____	F(2,68) = 0.37 p = 0.69
FQ	33.7 (20.0)	36.4 (20.3)	34.5 (21.3)	____	F(2,68) = 0.11 p = 0.90
ACQ (SD)	28.7(8.47)	29.4(11.6)	30.2(12.2)	____	F(2,68) = 0.11 p = 0.90
BSQ	45.2 (13.6)	50.1 (13.5)	43.7 (18.0)	____	F(2,68) = 0.91 p = 0.41

IE: interoceptive exposure

PDSS: Panic Disorder Severity Scale

FQ: Fear Questionnaire

ACQ: Agoraphobic Cognitions Questionnaire

BSQ: Body Sensations Questionnaire

### Factor analysis

We submitted all 17 data items (baseline, 71 patients; endpoint, 60 patients; 3-month follow-up, 47 patients; and 1-year follow-up, 30 patients) to an exploratory principal factor analysis and used a Promax rotation to extract oblique factors. The Kaiser Criterion as well as a visual inspection of the scree plot supported a four-factor solution.

Table 2 shows the rotated component matrix of the four-factor solution. The four-factor solution contained two items ('distorted vision' [item 8] and 'sweating' [item 14]) that failed to load onto any factor. The items that loaded on Factor I were 'numbness in limbs' (item 3), 'fingertip tingling' (item 4), 'other numbness' (item 5), and 'wobbly legs' (item 13). Therefore, Factor I was named "pseudoneurological fears." The items that loaded on Factor II were 'nausea' (item 9), 'butterflies in stomach' (item 10), 'knots in stomach' (item 11), and 'dry throat' (item 15). Therefore, Factor II was named "gastrointestinal fears." The items that loaded on Factor III were 'heart palpitations' (item 1), 'pressure in chest' (item 2), 'shortness of breath' (item 6), 'dizziness' (item 7) and 'lump in throat' (item 12). Therefore, Factor III was named "cardiorespiratory fears." The items that loaded on Factor IV were 'disorientation' (item 16) and 'disconnected from body' (item 17). Therefore, Factor IV was named "fears of dissociative feelings."

**Table 2:** Rotated factor loading of BSQ

BSQ items	Factor I "Pseudo-neurological fears"	Factor II "Gastrointestinal fears"	Factor III "Cardio-respiratory fears"	Factor IV "Fears of dissociative feelings"
3. Numbness in limbs	**1.08**	-0.11	0.33	-0.14
5. Other numbness	**0.94**	-0.02	-0.14	0.04
4. Fingertips tingling	**0.85**	-0.07	0.14	-0.33
13. Wobbly legs	**0.43**	0.34	-0.13	0.15
8. Distorted vision	0.34	0.22	0.05	0.29
10. Butterflies in stomach	-0.07	**0.94**	0.06	-0.18
9. Nausea	-0.08	**0.89**	-0.07	-0.02
11. Knot in stomach	0.03	**0.72**	-0.07	0.03
15. Dry throat	-0.06	**0.41**	0.29	0
6. Shortness of breath	-0.17	-0.07	**0.96**	0.04
1. Heart palpitations	0.03	0.01	**0.83**	-0.12
2. Pressure in chest	0.22	-0.05	**0.64**	-0.03
12. Lump in throat	0.18	0.19	**0.39**	0.1
7. Dizziness	0.05	0.14	**0.38**	0.24
14. Sweating	0.18	0.26	0.27	0.08
16. Disorientation	-0.05	-0.06	-0.05	**0.94**
17. Disconnected from body	-0.04	-0.09	0.02	**0.94**
Variance explained	47.1	9.4	7.6	6.4
Cronbach α	0.90	0.82	0.85	0.89

Items with a loading on a given factor of greater than 0.35 are set in boldface.

All the factors had Cronbach alpha coefficients greater than 0.75 and were therefore considered to be reliable.

### Correlations between degree of similarity of interoceptive exposure tasks and BSQ factor scores

Because of missing data, complete interoceptive exposure datasets were available for only 43 patients (Figure 1). We calculated the Spearman rank correlations between the degrees of similarity of each interoceptive exposure task to the patient's panic sensations and to each BSQ factor for each of these 43 subjects. (Table 3)

**Table 3:** Spearman rank correlations between BSQ factors and similarities reported in interoceptive exposures

Interoceptive exposures		BSQ Factors		
	
	I	II	III	IV
1. Hyperventilation	0.42**	0.26	0.20	0.10
2. Shaking head	0.42**	0.10	0.10	0.17
3. Putting head between legs	0.31*	0.17	0.02	0.17
4. Step-ups	0.22	0.20	0.22	-0.03
5. Holding breath	0.42**	0.26	0.14	0.14
6. Body tension	0.33*	0.01	-0.10	-0.19
7. Spinning	0.47**	0.28	0.30	0.44**
8. Breathing through a straw	0.25	0.39**	0.15	0.16
9. Chest breathing	0.50**	0.26	0.17	0.05

*p < 0.05, **p < 0.01

For Factor I (pseudoneurological fears), statistically significant correlations were obtained for several interoceptive tasks: 'hyperventilation', 'shaking head', 'holding breath', 'spinning' and 'chest breathing.' For Factor II (gastrointestinal fears), a statistically significant correlation was obtained for 'breathing through a straw.' For Factor III (cardiorespiratory fears), no statistically significant correlations were obtained. For Factor IV (fears of dissociative feelings), a statistically significant correlation was obtained for 'spinning.' 'Hyperventilation', 'shaking head', 'holding breath' and 'chest breathing' were therefore considered to reproduce pseudoneurological symptoms. 'Breathing through a straw' was considered to reproduce gastrointestinal symptoms. 'Spinning' was considered to reproduce both pseudoneurological and dissociative symptoms. None of the interoceptive exercises were considered to reproduce cardiorespiratory symptoms, such as palpitations and shortness of breath.

### Change after successful interoceptive exposure

Because the interoceptive exposure tasks selected as exercises were determined by the patient's ratings of their degrees of similarity to their own panic sensations, each patient had a different number of exposure tasks to perform. Table 4 shows the number of patients who selected each interoceptive task and the number of patients who completed each task successfully, i.e. who exhibited a decrease in their fear level by 30 points or more during the period in which the homework exercises were performed. Of the 43 patients whose records were available, 23 (53%) had their fear reduced by more than 30 points for one or more interoceptive exposure tasks, while 20 (47%) did not respond positively to any interoceptive exposure task.

**Table 4:** Number of patients who undertook each interoceptive exercise and who experienced areduction in fear by more than 30 points

	Patients who undertook the task	Patients who completed the task successfully
1. Hyperventilation	17	9
2. Shaking head	10	3
3. Putting head between legs	5	4
4. Step-ups	11	5
5. Holding breath	15	6
6. Body tension	5	0
7. Spinning	15	8
8. Breathing through a straw	14	4
9. Chest breathing	12	8

We calculated the BSQ factor scores before and after the treatment program for each patient who completed each task with success and then tested for significant changes. Tables 5, 6, 7 and 8 show the four interoceptive exercises that produced significant reductions in the BSQ factor scores, according to the Wilcoxon test.

Among the patients for whom 'hyperventilation' was effective, significant changes in all factors were observed. Among the patients for whom 'holding breath' was effective, a significant change in pseudoneurological fears was observed. Among the patients for whom 'spinning' was effective, significant changes in pseudoneurological, gastrointestinal and cardiorespiratory fears were observed. Among the patients for whom 'chest breathing' was effective significant changes in pseudoneurological, gastrointestinal and cardiorespiratory fears were observed.

'Shaking head', 'spinning' and 'breathing through a straw' were considered to represent pseudoneurological, dissociative and gastrointestinal symptoms, respectively. However, among the patients who performed the 'shaking head' and 'breathing through a straw' exercises, no significant changes between the baseline and endpoint scores were seen for any of the factors. In patients who performed the 'spinning' exercise, no significant change in the baseline and endpoint scores was seen for the 'fears of dissociative feelings' factor.

**Table 5:** BSQ factor scores before and after treatment among those who successfully completed the'hyperventilation' task (n = 9)

		Baseline		Endpoint		Wilcoxon test	
		Mean	SD	Mean	SD	Z	p

Factor I	Pseudoneurological fears	2.75	1.19	1.14	0.22	-2.52	0.012
Factor II	Gastrointestinal fears	2.47	0.74	1.28	0.32	-2.68	0.007
Factor III	Cardiorespiratory fears	2.91	0.78	1.6	0.35	-2.68	0.007
Factor IV	Fears of dissociative feeling	3.89	1.19	2.11	1.11	-2.55	0.011

**Table 6:** BSQ factor scores before and after treatment among those who successfully completed the'holding breath' task (n = 6)

		Baseline		Endpoint		Wilcoxon test	
		Mean	SD	Mean	SD	Z	p

Factor I	Pseudoneurological fears	2.12	0.97	1.37	0.68	-2.03	0.04
Factor II	Gastrointestinal fears	2.12	0.8	1.54	0.48	-1.82	0.06
Factor III	Cardiorespiratory fears	2.7	0.61	2.33	1.06	-1.09	0.27
Factor IV	Fears of dissociative feeling	2.83	1.12	2.16	1.57	-0.95	0.34

**Table 7:** BSQ factor scores before and after treatment among those who successfully completed the'spinning' task (n = 8)

		Baseline		Endpoint		Wilcoxon test	
		Mean	SD	Mean	SD	Z	p

Factor I	Pseudoneurological fears	3.09	1.23	1.56	0.63	-2.38	0.01
Factor II	Gastrointestinal fears	2.37	0.81	1.5	0.46	-2.37	0.01
Factor III	Cardiorespiratory fears	3.12	0.86	1.92	0.88	-2.2	0.02
Factor IV	Fears of dissociative feeling	3.43	1.29	2.37	1.27	-1.29	0.19

**Table 8:** BSQ factor scores before and after treatment among those who successfully completed the'chest breathing' task (n = 8)

		Baseline		Endpoint		Wilcoxon test	
		Mean	SD	Mean	SD	Z	p

Factor I	Pseudoneurological fears	3.25	1.05	1.65	0.74	-2.37	0.01
Factor II	Gastrointestinal fears	2.68	0.98	1.59	0.61	-2.37	0.01
Factor III	Cardiorespiratory fears	3.3	0.95	2.32	0.95	-2.37	0.01
Factor IV	Fears of dissociative feeling	3.75	1.43	2.75	1.6	-1.7	0.08

## Discussion

The main findings of the present study are as follows.

(1) Four symptom factors emerged through factor analyses of the BSQ data; these factors were named pseudoneurological fears, gastrointestinal fears, cardiorespiratory fears, and fears of dissociative feelings. This factor structure is roughly in line with the findings of a previous study that described a three-factor structure consisting of somatic fears, cardiac fears and psychosensual fears [22], where their first factor corresponds with our gastrointestinal fears, their second factor corresponds with our pseudoneurological and cardiorespiratory fears, and their third factor corresponds with our fears of dissociative feelings. Thus, the fears of physical sensations associated with panic attacks can apparently be captured using these three or four perspectives.

(2) Among the nine interoceptive exposure tasks, 'hyperventilation', 'shaking head', 'holding breath' and 'chest breathing' were considered to reproduce pseudoneurological symptoms; 'breathing through a straw' was considered to reproduce gastrointestinal symptoms; and 'spinning' was considered to reproduce both pseudoneurological and dissociative symptoms. On the other hand, none of the interoceptive exercises were considered to reproduce cardiorespiratory symptoms.

Some of the observed correlations between the interoceptive exposure tasks and the BSQ factors were as expected, while others were unanticipated. For example, 'hyperventilation' and 'chest breathing' reproduced pseudoneurological symptoms and 'spinning' reproduced dissociative feelings, as expected. On the other hand, the inability of the 'step-ups' and 'breath holding' exercises to elicit cardiorespiratory symptoms was surprising. This result appears to contradict that reported by Antony et al. [5], who reported that cardiorespiratory symptoms were strongly elicited by exercises like 'holding breath', 'hyperventilation', 'breathing through straw', 'running on the spot' and 'sitting facing a heater.' However, Antony et al. recorded the symptoms elicited by each exercise, while we correlated the similarities between each exercise and interoceptive fears, as measured by the BSQ. Consequently, the palpitations produced after the 'step-ups' exercise or the shortness of breath produced after the 'holding breath' exercise may have been simply regarded as normal physiological responses, causing the patients not to perceive these responses as being similar to their own panic sensations.

Likewise, 'breathing through a straw' was designed to elicit symptoms of breathlessness. In this study, however, 'breathing through a straw' reproduced gastrointestinal symptoms. Gastrointestinal symptoms like nausea and abdominal distress may have been elicited as a result of the patients placing straws in their mouths.

(3) In the patients who performed the 'hyperventilation', 'holding breath', 'spinning' and 'chest breathing' exercises, a significant improvement in the BSQ Factor I (pseudoneurological fears) score was observed. Therefore, the interoceptive exercises were useful for reducing fears of pseudoneurological sensations. On the other hand, 'spinning' and 'breathing through a straw' were considered to reproduce dissociative and gastrointestinal symptoms, respectively, but no significant differences between the baseline and endpoint BSQ scores for Factor II (gastrointestinal fears) or Factor IV (fears of dissociative feelings) were observed.

Previous reports have suggested that an interoceptive exposure component is essential to effective CBT programs [23-25]. In a randomized controlled trial (RCT), interoceptive exposure (10 items) and breathing retraining were compared. When performed in conjunction with cognitive restructuring and in vivo exposure, the two exercises were equally effective according to several measures, but interoceptive exposure was more effective than breathing retraining in terms of panic frequency, overall severity, and functioning [7]. In another RCT, interoceptive exposure (5 items) and breathing retraining was as effective in reducing panic and agoraphobic symptoms over short-term and long-term treatment periods as was in vivo exposure only and combined interoceptive and in vivo exposure with breathing retraining [9]. In a case study, interoceptive exposure alone was found to be effective in reducing panic, panic-related fears, and general anxiety [8]. The present study confirmed that interoceptive exposure tasks like 'hyperventilation', 'holding breath', 'spinning' and 'chest breathing' have significant effects on the fears of physical sensations experienced by patients with the panic disorder.

The present study has a few possible limitations. First, the sample size was rather small, especially for each interoceptive exposure task. Thus, small yet important effects of some interoceptive exposure tasks may have been missed. Second, the study did not include a control group, and it relied on within-group changes to examine the effects of interoceptive exposure. Therefore, the individual effects of the shared treatment components (breathing retraining, cognitive restructuring and in vivo exposure) could not be isolated. Our treatment program followed Andrews et al.'s protocol [17] and emphasized controlled breathing. Thus, it seems reasonable to suppose that our program might reduce panic symptoms related to respiration more than those related to other functions. The study by Beck et al. [8] is the only report to examine the effects of interoceptive exposure when used alone. Also, because each interoceptive exposure task was performed in conjunction with other tasks, the results for a given task might have been influenced by the performance of other tasks. Lastly, even when a certain task targets certain interoceptive fears, reducing one factor in the fear network may have generalized effects on the other fears.

As far as we are aware, the present study is the first report to confirm that interoceptive exposure tasks can reproduce specific fears of physical sensations and that each interoceptive exposure task affects the fear of physical sensations. Feared physical sensations differ from one individual to another, and the interoceptive exposure tasks to be performed are selected on the basis of their similarity to a patient's unique panic sensations. Although the efficacy of interoceptive exposure has been confirmed, the efficacies of individual interoceptive exposure tasks have not been previously studied. This study was an exploratory report that lacked a control group, clearly limiting the interpretability of this investigation. However, these findings provide a basis for the reconfirmation of each interoceptive exposure task. A more systematic study of the mechanisms of action of interoceptive exposure using a larger sample that can be followed for a longer interval after interoceptive exposure is needed. Ideally, the effects of interoceptive exposure tasks should be studied separate from those of breathing retraining, cognitive restructuring, in vivo exposure and medications.

## Conclusion

In conclusion, among the nine interoceptive exposure tasks, 'hyperventilation', 'holding breath', 'spinning' and 'chest breathing' were regarded as representing physical sensations and had a significant effect on reducing the fears of physical sensations experienced by patients with panic disorder. Further study of interoceptive exposure exercises may be necessary. Interoceptive exposure tasks that reproduced certain fears of physical sensations but produced no significant reduction in fear may need to be improved, while new exercises to reproduce cardiorespiratory symptoms need be devised.

## Competing interests

The author(s) declare that they have no competing interests.

## Authors' contributions

KL was the primary investigator; KL, Y.Noda, Y.Nakano, SO, YK, TF and TAF performed the clinical investigation (diagnosis, treatment, assessment and follow-up phases); TAF participated in the design of the study and supervised the overall conduct of the study. All authors approved the final manuscript.

## Pre-publication history

The pre-publication history for this paper can be accessed here:


